# Effects of Virtual Reality training on medical students’ learning motivation and competency

**DOI:** 10.12669/pjms.35.3.44

**Published:** 2019

**Authors:** Mian Usman Sattar, Sellappan Palaniappan, Asiah Lokman, Atif Hassan, Nauman Shah, Zurabia Riaz

**Affiliations:** 1 *Mian Usman Sattar, PhD, (Informatics) Student., School of Science and Engineering, Malaysia University of Science and Technology, Malaysia*; 2 *Dr. Sellappan Palaniappan, PhD. Dean School of Science and Engineering, School of Science and Engineering, Malaysia University of Science and Technology, Malaysia*; 3 *Dr. Asiah Lokman, PhD. Assistant Professor, School of Science and Engineering, Malaysia University of Science and Technology, Malaysia*; 4 *Dr. Atif Hassan, Post- Doctorate. Director of School of Professional Advancement, Department of Information Systems, University of Management and Technology, Lahore, Pakistan*; 5 *Dr. Nauman Shah, PhD. Chairperson of Information Systems Department, Department of Information Systems, University of Management and Technology, Lahore, Pakistan*; 6 *Zurabia Riaz, MS. ERP Officer, Department of Information Systems, University of Management and Technology, Lahore, Pakistan*

**Keywords:** Virtual reality (VR), video based learning (VBL), text based learning (TBL)

## Abstract

**Objectives:**

To determine the need of contemporary immersive approaches (Virtual Reality) in teaching and training at medical sector. The main objective of this study was to explore the effects of text, video and immersive technologies learning methodologies for participants’ learning in public and private medical colleges and universities of Pakistan.

**Methods:**

In this quantitative research 87 medical students of 4^th^ year from three public and five private medical colleges and universities participated. A laparoscopy operation was selected in consultation with senior medical consultants for this experiment. The experimental material was arranged in virtual reality, video and text based learning. At completion of each of which, participants completed a questionnaire about learning motivation and learning competency through the different mediums.

**Results:**

Statistical t-test was selected for the analysis of this study. By comparing the mean values of virtual reality, video, and text based learning methodologies in medical academics; result of virtual reality is at top of others. All performed model are statistically significant (P=0.000) and results can be applied at all population.

**Conclusion:**

Through this research, we contribute to medical students learning methodologies. In medical studies, both theoretical and practical expertise has a vital role, while repetition of hands-on practice can improve young doctors’ professional competency. Virtual reality was found best for medical students in both learning motivation and learning competency. Medical students and educationist may select virtual reality as new learning methodology for curriculum learning.

## INTRODUCTION

In the last decade, the rapid innovation in the technological world has had a major impact on the human-computer interaction (HCI). The education sector has also realized its significance and has made various attempts to use it to their advantage.^1^ Various technologies have been introduced to enhance the learning, engagement of the students, and their assessments, aiming to enrich the quality of education.^2^,^3^ The motivation of students to effectively learn plays an effective role is one of such key priorities for educational systems. This paper explores the effects of using virtual reality simulations to train medical students on their motivation to learn.

Virtual Reality (VR) technologies have transformed the landscape of HCI in recent years by offering new and unique viewpoints on the core goals of training and education. VR creates a sense of immediacy and control through its immersive display which puts the user into a simulated environment that looks and feels to a certain extent like the real world. This immersion, in turn, engages the user by attaining their attention to the fullest resulting in better learning outcomes.^4^

This trend has also taken over the education sector within the medical field with virtual reality applications developed to teach medical students a range of topics from anatomy^5^ to surgery.^6^ Moreover; a virtual reality environment gives the patients a more sense of immersion mentally preparing them for real life scenarios.^7^

### Virtual reality based learning

There is a wide range of trainings and counseling sectors which are getting benefits of text, video and virtual reality including medical, surgery, construction specialists, law enforcement, military person-to-person training service and maintenance, marketing, serious games, speak therapy, training of architecture, engineering or and sports.^8^,^9^

Due to increasing complexity and high range of integrated task medical processes became complex entities for the system. Thus it is necessary to train the medical staff with high demand revival skill.^8^ Conventional training facilities involved traveling expenses, traveling time, trainers cost, running cost and most important these can be delivered in synchronous mode. Both trainer and trainee should present at same place at a time.^10^ VR provides virtual simulation though sound in immersive environment. VR training is being used in such complex domains and situations which cannot be created to experience physically.^11^

Medical Reality application is having multiple medical related business associations which endorsed this application, module quality and ensure curriculum.^11^

### Medical Counseling

Virtual reality is being used in psychiatry for counseling of patients including depression, bulimia, anorexia nervosa, and agoraphobia, fear of public speaking, obesity, claustrophobia, and acrophobia. For all these specialized tasks, hardware of head mounted 3-D equipment and simulated trainings were designed to evoke motivation among virtual environment and subject.^12^ These environments were created against the natural situation including avatars, their movement, 3-D sound system and facial expression. Through this flexibly modified environment, specialized scenarios as per unique clinical requirements could be created so that accurate sensorial engagement will be established.^13^

### Surgical Training

VR is also in fashion in surgery trainings specially rat dissection, basic suturing and knot tying, virtual anatomy laboratory, arthroscopy, interventional radiography and cardiology, maxillofacial surgery, laparoscopy, peritoneal lavage, epidural injection, neurosurgery, trauma training, and 3-D radiologic clinic etc.^6^

Simulators depict trainings in laparoscopy for general surgery, endoscopic surgery, arthroscopy, neuroendoscopy, cystoscopy and bronchoscopy.^14^ Special software was selected to mock-up laparoscopic instruments and tissues to build up a nearest to real time VR training environment. Surgical infrastructure used electromagnetic racking system were connected to real time surgical equipment so their exact compliment could be created in virtual world.^15^

In haptic structure, devices were designed to give the force feedback of every observance of patient tissue touched. Variety of equipment included head mounted display to laptop and desktop systems used for displaying process. These systems were also aimed to produce quantitative feedback, growth in skill, competency and consistent ways to access skill set.^16^ Therefore the main objective of surgical simulations was to develop a VR environment for surgical trainings, evaluation of user’s performance and competency testing.^17^ All these studies were expressive and were proposed usage of disruptive technologies in medical for technological novelty in VR in both applications and equipment. No comparative study was conducted for the efficiency, effectiveness and acceptability of the virtual reality in medical. No study explained the adaptability of routine use of simulation environment.^18^

### Contemporary Operating Theater Training

Operation Theater is known as battle field for the surgeons. Complication and tricky situations can only be handled through skill set of the surgeon and experience. Ideally, VR based trainings improves performance in real scenario and help in increasing the skill set for operation room thus reduce the complication rate correlated with inexperience.^6^

The effect at efficiency of simulation training has not yet been measured in the surgery process. But in case of Pakistan, medical colleges are facing serious deficiency of equipment, operation rooms and even space. Through virtual reality proficiency based trainings, medical colleges both public and private can train their medical students for result oriented benchmarking which can be measured through related performance metrics.^19^

This simulation based trainings program could be an effective way to train the medical students in their professional skills and is widely used in ophthalmology trainings.^19^,^20^ In distinction to the time and repetition based training, proficiency based training can make sure about the surgeon who meet the required benchmark in VR training will ultimately allowed to operate the patient.^20^

### Research Hypothesis

For this study different hypothesis has been proposed. All hypotheses tested through experiment to find out the reliability of the study.

**H1:** Learning motivation and learning competency of participants through VR will relatively be higher than text based learning methodology.

**H2:** Learning motivation and learning competency of participants through VR will relatively be higher than video based learning methodology.

**H3:** Learning motivation and learning competency of participants through videobased learning methodology will be relatively higher than text based learning methodology.

## METHODS

Text, video and VR methodology of learning is selected for experiment. Same teaching material for laparoscopy operation is prepared in selected learning methodologies. Participants of the study were medical students of public and private sector medical colleges and universities of Pakistan. All participants experienced all selected learning methodologies in random order so that that effect of learning motivation and learning competency can be evaluated.

### Experimental setup

Participants from three public and five private medical colleges and universities contributed in this study carried over a period of four months after acquiring the ethics approval IPS/PIN/P18030005 The study was setup at each of the university and participants were recruited voluntarily through announcements. Medical Realities application by Medical Realities Ltd was selected for virtual reality and video based learning experience. The Universities included Sharif Medical and Dental College, Lahore, Faisalabad Medical University, Faisalabad, Sheikh Zayed Medical College, Rahim Yar Khan, Lahore Medical and Dental College, Lahore, Allama Iqbal Medical College, Lahore and Fatima Memorial College of Medicine and Dentistry, Lahore. Overall 87 volunteers from eight institutions participated in this research study; 49 male and 38 females with mean age 22.1 years. English was the primary language of communication and all resources were designed in English.

**Fig.1:**
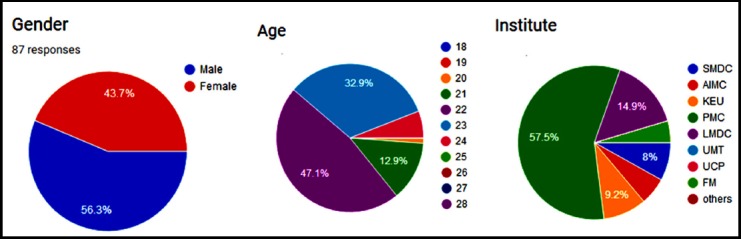
Respondent Demographics (Gender, Age, Institute)

### Stage One

In stage one; first author gives out instructions, outcome and brief about risks of using VR. Proper demonstration was provided about Medical Reality application navigation. This navigation demonstration helped respondents to reach smoothly at required module of laparoscopy 360 degree.

After completion of this briefing and demonstration stage each respondent was asked to do the VR learning experience by using Samsung Oculus head mounted display unit. Participants are advised to complete at least four minutes in VR environment for better user experience. Respondents gave their feedback at the end of experiment.

### Stage Two

In stage two; first author gave briefing about video based learning (VBL), its effects and outcomes. The respondents were then asked to view a video of laparoscopy operation. After completion of video the participants were asked to fill a questionnaire about the learning motivation and learning competency through video based learning methodology.

**Fig.2:**
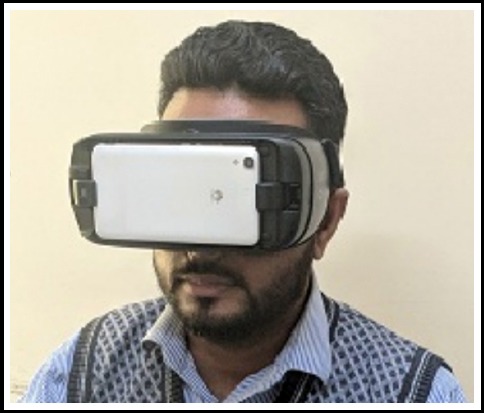
MBBS 4th year student experiencing VR.

### Stage Three

In stage three; first author gave briefing about text based learning methodology, its effects and outcomes. Then respondents were asked to read a printed text file of about one and half page. Text file have same information of laparoscopy operation. Average time of reading was around 86 seconds. After completion of text based user experience they were asked to complete a questionnaire about learning motivation and learning competency through text base learning methodology.

### Experimental Outcomes

In this study we evaluate effect of learning motivation and learning competency of medical students through text, video and VR learning methodologies. The study was carried out through pre-validated questionnaire. Tool of learning motivation and learning competency was adapted from intrinsic motivation inventory (IMI). 21,22

**Fig.3:**
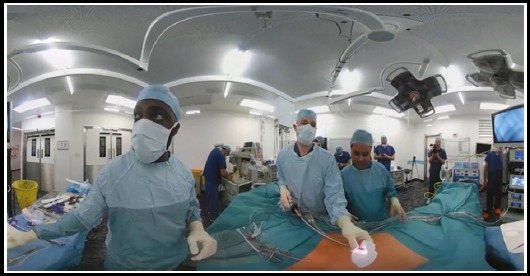
Video view of laparoscopy.

The questionnaire covers two categories; learning motivation and learning competency. Both categories have four questions and respondent used Likert scale from one to seven for feedback, where one to seven scale with one implying ‘strongly disagree’ and seven being ‘strongly agree’. Average each participant spent four minutes for VR experience, 1.49 minutes for text and 2.29 minutes to video.

Following abbreviation are being used VRLM for VR learning motivation, VRLC for VR learning competency, VBLM for video based learning motivation, VBLC for video based learning competency, TBLM for text based learning motivation and TBLC text based learning competency.

In comparison, it was observed that the VR learning motivation and competency both had higher mean values than the text based learning motivation and competency, hence proving H1. Similarly, the VR learning motivation and competency had higher mean values in comparison to the video based learning values, hence proving H2. Moreover, we see that the mean values for learning motivation and competency are higher for text based learning in comparison to the video-based, which disapproves H3.

**Fig.4:**
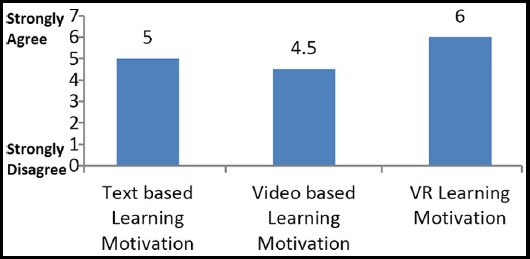
Comparison of Learning Motivation.

**Fig.5:**
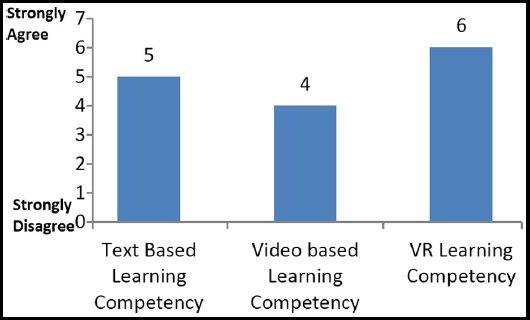
Comparison of Learning Competency.

Fig. 4 and 5 shows the comparison of all three learning methodologies together which were selected for this study. Learning motivation and learning competency through VR is higher than both of the other learning methodologies. Moreover, text based learning motivation and competency is better than that of video based learning.

## DISCUSSION

Paired sample t-test was selected for hypothesis verification. All assumptions of t-test were verified before applying test in SPSS. Mean of the difference (VRLM-TBLM) which is 0.63506 with standard deviation 0.88001. The test-statistic value is 6.731 with p-value 0.000 which is less than 0.05. Mean (VRLM) is significantly differing from Mean (TBLM). Mean of the difference (VRLC-TBLC) which is 0.69540 with standard deviation 0.93807. The test-statistic value is 6.914 with p-value 0.000 which is less than 0.05. Mean (VRLC) is significantly differing from Mean (TBLC).

Mean of the difference (VRLM-VBLM) which is 1.25000 with standard deviation 1.01729. The test-statistic value is 11.461 with p-value 0.000 which is less than 0.05. Mean (VRLM) is significantly differing from Mean (VBLM).Mean of the difference (VRLC-VBLC) which is 1.64655 with standard deviation 1.14599. The test-statistic value is 13.401 with p-value 0.000 which is less than 0.05. Mean (VRLC) is significantly differing from Mean (VBLC).

Mean of the difference (TBLM-VBLM) which is 0.61494 with standard deviation 1.18788. The test-statistic value is 4.829 with p-value 0.000 which is less than 0.05. Mean (TBLM) is significantly differing from Mean (VBLM). Mean of the difference (TBLC-VBLC) which is 0.95115 with standard deviation 1.18788. The test-statistic value is 6.715 with p-value 0.000 which is less than 0.05. Mean (TBLC) is significantly differing from Mean (VBLC).

As per above results and discussions the overall results of this experiment are significant and can be implemented to population. Similar to previous studies, the study concludes that students have a positive and optimistic view of virtual reality usage in academic learning process.^23^ Immersive technologies encourage students to become active learners especially to understand complex concepts.^24^

## CONCLUSIONS

By observing experimental results, data analysis and discussions hypothesis H1, H2 are proved but H3 was disproved. Through this research we have tried to contribute in medical students learning methodology. In medical studies, both theoretical and practical expertise are having vital role. Repetition of practical can improve young doctor’s professional competency. Immersive technologies were found best for medical students in both learning motivation and learning competency. Medical students and educationist may introduce VR and new learning methodology for curriculum learning. Furthermore, longitudinal research and addition of more dimensions in medical sciences can improve quality of research.

### Author’s Contribution

**MUS:** Accountable for all aspects of the work in ensuring that questions related to the accuracy and data analysis.

**SP:** Final approval of the version to be published.

**AL:** Critically revising the important intellectual content.

**AH:** Performed acquisition, analysis, and interpretation of data for the work.

**NS:** Drafted the work and assure the integrity of any part of the work to be appropriately investigated and resolved.

**ZR:** Provide substantial contributions to the conception.
